# Editorial: Soil microbial communities to promote suppressiveness against soil-borne pathogens and diseases

**DOI:** 10.3389/fmicb.2025.1677158

**Published:** 2025-09-16

**Authors:** Jose Ignacio Marín-Guirao, Dongdong Yan, Francesco Di Gioia, Massimo Pugliese, Jesus D. Fernandez-Bayo

**Affiliations:** ^1^Andalusian Institute of Agricultural and Fisheries Research Training (IFAPA) La Mojonera, Almería, Spain; ^2^Chinese Academy of Agricultural Sciences (CAAS), Beijing, China; ^3^The Pennsylvania State University (PSU), University Park, PA, United States; ^4^University of Turin – Agroinnova, Grugliasco, Italy; ^5^Department of Soil Science and Agricultural Chemistry, University of Granada, Granada, Spain

**Keywords:** suppressive soils, soil fumigation alternative, beneficial soil microbes, sustainable agricultural practices, soil biosolarization

## Introduction

The phaseout of methyl bromide, stringent regulations on fumigant application, and the consumer demand for safer food are leading farmers toward the adoption of more sustainable soil management practices for the control of soil-borne pathogens (Rosskopf et al., 2024). In this context, organic amendment-based approaches such as soil biofumigation, biosolarization, and anaerobic soil disinfestation have emerged as promising alternatives to the use of synthetic soil fumigants, especially for their ability to suppress soilborne pathogens while preserving soil health and the environment. However, their widespread adoption is still limited by technical and sustainability constraints, such as the variability in efficacy across soils and climates, relatively high costs, amendment type, and use of impermeable plastic barriers. Given these limitations, new approaches aimed at enhancing natural soil suppressiveness and shifting soil microbial communities against soil-borne pathogens have gained increasing attention. Soil microorganisms, whether native or introduced *ex novo*, play a pivotal role in providing ecosystem services that can contribute to this goal by enhancing both soil and plant health. Thus, harnessing this biological potential offers a promising way to reduce agrochemical dependency and increase the resilience of cropping systems.

The main aim of this Research Topic was to expand knowledge on how soil management practices influence microbial communities and their ability to suppress soil-borne pathogens. The contributions span both fundamental understanding and practical implications, addressing the effects of soil amendments, microbial community shifts associated with disease suppression, interactions with plant defense mechanisms, and the overall effects on soil health and crop yield. Together, the 12 articles included reflect a growing body of evidence that shows how soil microbial communities can contribute to

the sustainable management of soil-borne pathogens and enhance soil health and productivity (Table 1).

**Table 1:** Summary of beneficial organisms added to the soil or agronomic practices, target pathogens and target crops included in the Research Topic.

**Beneficial organisms added to the soil or agronomic practices**	**Target pathogen**	**Target crop**	**References**
*Trichoderma* sp	*Fusarium oxysporum* f. sp. *cubense* subtropical race 4 (Foc-STR4)	Banana (*Musa acuminata*)	Correa-Delgado et al.
*Bacillus velezensis* NT35	*Ilyonectria robusta*	*Panax ginseng*	Li et al.
*Bacillus velezensis* BF-237	Wheat crown rot (WCR). *Fusarium* sp	Wheat	Wang Y. et al.
*Serratia marcescens and Paenibacillus polymyxa*	NA	*Cajanus cajan*	Liu et al.
*Nostoc punctiforme*	*Heterodera glycines Ichinohe*	Soybean [Glycine max (L.) Merr.]	Yin et al.
Application of biochar, lime or both	*Ralstonia solanacearum*	Tobacco (*Nicotiana tabacum* L.)	Bian-hong et al.
Bio-organic fertilizers enriched with *T. asperellum, B. amyloliquefaciens*, and *B. subtilis*	Unidentified root disease	*Codonopsis pilosula*	Huang et al.
Native bacteria from biosolarized soil with fresh sheep manure	*Colletotrichum gloeosporioides, F. proliferatum*, F. *oxysporum* f. sp. *radicis-cucumerinum, Botrytis cinerea* and *Phytophthora capsici*	Tomato (*Solanum lycopersicum* L)	Marín-Guirao and de Cara-García
Functional rhizosphere microbiomes associated with plant genetic factors	Fungal plant pathogens. I.e. *F. oxysporum*	*Panax notoginseng*	Shi et al.
Fumigation with allyl isothiocyanate	NA	Peppers *(Capsicum annuum L.)*	Tian et al.
Crop rotation	*F. oxysporum*	*Vanilla (Vanilla planifolia)* *black pepper (Piper nigrum L.)* *sweet rice tea (Strobilanthes tonkinensis Lindau)*	Hong et al.
Intercropping	NA	*Torreya grandis cv Merrillii* *Polygonatum sibiricum*	Wang Q. et al.

## Outline of contributions

Several contributions emphasized the use of native beneficial microbes with biocontrol potential, while underscoring the importance of single and mixed microbial inoculants in disease suppression strategies. Correa-Delgado et al. provided a detailed inventory of *Trichoderma* spp. from banana rhizospheres, identifying 10 species, six of which were reported for the first time in the Canary Islands, along with two putative novel taxa. Their findings revealed distinct distribution patterns across agroecosystems and a strong correlation with soil chemical properties, particularly pH and phosphorus levels. This comprehensive survey offers a foundation for the development of future biocontrol strategies against *Fusarium oxysporum* f. sp. *cubense* subtropical race 4. Li et al. demonstrated that *Bacillus velezensis* NT35, a strain isolated from the rhizosphere soil of *Panax ginseng*, exhibited antifungal activity and enhanced resistance to *Ilyonectria robusta* by modulating the expression of defense-related genes and shaping the rhizosphere microbiota. Similarly, focusing on *Bacillus velezensis* BF-237, a strain isolated from wheat rhizosphere soils, Wang Y. et al. demonstrated its efficacy in reducing wheat crown rot (WCR) severity and in supporting beneficial microbes under pathogen pressure. Furthermore, functional predictions suggest that microbial communities adapt to WCR by enhancing signaling pathways and decreasing anabolic activity. Conversely, a native mixed inoculant composed of *Serratia marcescens* and *Paenibacillus polymyxa* promoted *Cajanus cajan* growth (Liu et al.). This inoculant caused more pronounced changes in the soil fungal community than in bacterial communities, indicating that soil fungi are more sensitive to the inoculation of non-native biocontrol agents. Changes in the abundance of both beneficial and potentially pathogenic fungi were detected, alongside improved nutrient availability and plant development. Finally, Yin et al. reported that the cyanobacterium *Nostoc punctiforme* mitigated the soybean cyst nematode by indirectly reshaping the rhizosphere microbiota, rather than directly triggering host-defense mechanisms. The inoculation altered bacterial and fungal community composition in the soybean rhizosphere, increasing the relative abundance of taxa with potential nematicidal activity.

Other studies explored the interaction between soil amendments and microbial communities in the context of disease suppression. Bian-Hong et al. reported that lime, biochar, and a combination of the two reduced the tobacco bacterial wilt caused by *Ralstonia solanacearum* and improved tobacco yield in acidified soils. Biochar and lime-biochar treatments improved soil pH, and nutrient availability, while reducing acidity and bulk density. All treatments expanded niche breadth, enhanced positive microbial interactions, and intensified negative interactions involving *R. solanacearum*. Huang et al. showed that bio-organic fertilizers, based on prickly ash seed meal enriched with beneficial microorganisms such as *T. asperellum, B. amyloliquefaciens*, and *B. subtilis*, significantly reduced root rot and improved *Codonopsis pilosula* yield and quality compared to synthetic fertilizers. These effects were attributed to modifications in the structure of the rhizosphere bacterial community, enhanced stability of the microbial network, and enrichment of key taxa such as *Microlunatus, Rubrobacter*, and *Nakamurella*. Functional analyses indicated that bacterial signal transduction and amino acid metabolism may play a central role during the early and mid-growth stages. The study by Marín-Guirao and de Cara-García, conducted over two seasons in an organic Mediterranean tomato greenhouse, demonstrated that native bacterial diversity, shaped by biosolarization with fresh sheep manure, may enhance natural suppressiveness but also interfere with the performance of introduced biocontrol agents. A notable finding of the study was the high prevalence of native bacteria with antagonistic properties, primarily *Streptomyces* spp. and *Bacillus* spp., which inhibited the growth of both pathogenic and beneficial fungi sourced from commercial products.

Crop genotype and previous cultivation practices have also been identified as major factors influencing the composition and assembly of soil and rhizosphere microbial communities. Shi et al. demonstrated that the deterministic assembly of biocontrol-associated microbial communities in the rhizosphere of *Panax notoginseng* was particularly evident during the third year of root development and was influenced by plant genetic pathways. Transcriptomic analyses revealed that genes involved in protein export, alkaloid and amino acid biosynthesis, along with associated transcription factors, contributed to the recruitment of beneficial microbial taxa. Tian et al. showed that allyl-isothiocyanate fumigation significantly alters soil microbial diversity and composition, notably promoting Actinomycetota and suppressing Pseudomonadota. However, its effects on endophytic bacterial communities differed among pepper genotypes, highlighting the complex interactions among fumigation, soil microbiota, and plant internal microbiomes.

Finally, crop rotation and intercropping have proven to be effective strategies for disease management linked to soil microbiome modulation. Hong et al. showed that rotating vanilla with pandan or sweet rice tea significantly reduced *Fusarium* wilt by decreasing *F. oxysporum* abundance, enhancing fungal diversity, and enriching beneficial microbial taxa. In addition, the raised soil pH along with the altered microbial communities, was directly associated with pathogen suppression and enhanced vanilla disease resistance. Wang Q. et al. demonstrated that intercropping *Torreya grandis* with *Polygonatum sibiricum* enhanced soil microbial diversity, reduced the relative abundance of fungal genera, including potential soil-borne pathogens (e.g., *Cladosporium, Fusarium, Neocosmospora*), and enriched microbial groups involved in carbon and nitrogen cycling. These findings further support the role of diversified plant systems in fostering microbial diversity and pathogen suppression.

## Conclusion

The studies in this Research Topic offer valuable frameworks for transitioning to more sustainable alternatives in the management of soil-borne pathogens. Together, they underscore the importance of managing soil microbial communities as a cornerstone of any sustainable strategy aimed at improving soil and plant health. These contributions provide compelling evidence that biotic and abiotic interventions, including microbial inoculants, organic amendments, genotype selection, and cropping system diversification, can enhance pathogen suppression by promoting beneficial taxa and restructuring microbial networks. Nevertheless, the complexity of microbial interactions, underlying mechanisms and key microbial traits in disease-suppressive soils remains largely elusive. Therefore, recognizing that each agroecosystem presents unique conditions and challenges, tailored approaches are required for the successful generation of disease-suppressive soils.
